
JOURNAL INFORMATION
==============================
NLM Title Abbreviation: J High Energy Phys
Journal ID: J High Energy Phys
NLM Journal ID: 101668727

Journal of High Energy Physics

ISSN: 1126-6708
EISSN: 1029-8479

ARTICLE INFORMATION
==============================
PMCID: PMC8827381
PMID: 35212684
DOI: 10.1007/JHEP03(2016)001
Article ID: 3258
Article version: 1
Subjects: Erratum

Erratum to: Elements of a theory for multiparton interactions in QCD

Diehl Markus markus.diehl@desy.de
1
Ostermeier Daniel Daniel.Ostermeier@physik.uni-regensburg.de
2
Schäfer Andreas Andreas.Schaefer@physik.uni-regensburg.de
2
1 grid.7683.a 0000000404920453 Deutsches Elektronen-Synchroton DESY, 22603 Hamburg, Germany
2 grid.7727.5 0000000121905763 Institut für Theoretische Physik, Universität Regensburg, 93040 Regensburg, Germany
Electronic publication date: 2016 Mar 1
Print publication date: 2016
Volume: 2016
Issue: 3
Electronic Location ID: 1
Received 2016 Feb 16; Accepted 2016 Feb 16
Copyright: © The Author(s) 2016
License: Open AccessThis article is distributed under the terms of the Creative Commons Attribution 4.0 International License (https://creativecommons.org/licenses/by/4.0), which permits use, duplication, adaptation, distribution, and reproduction in any medium or format, as long as you give appropriate credit to the original author(s) and the source, provide a link to the Creative Commons license, and indicate if changes were made.
License URL: https://creativecommons.org/licenses/by/4.0/

Erratum for: 10.1007/JHEP03(2012)089
issue-copyright-statement: © SISSA, Trieste, Italy 2016

==============================
Open Access

This article is distributed under the terms of the Creative Commons Attribution License (CC-BY 4.0), which permits any use, distribution and reproduction in any medium, provided the original author(s) and source are credited.

Erratum to: JHEP03(2012)089

ArXiv ePrint: 1111.0910

